# A Scoping Review of Therapy Provision Under the National Disability Insurance Scheme: Provider‐Identified Barriers and Facilitators

**DOI:** 10.1111/jar.70278

**Published:** 2026-07-10

**Authors:** M. D. Marveggio, D. S. Dorstyn, D. Turnbull, A. Thirumanickam

**Affiliations:** ^1^ College of Education, Behavioural and Social Sciences, School of Psychology Adelaide University Adelaide Australia; ^2^ College of Health, School of Allied Health and Human Performance Adelaide University Adelaide Australia; ^3^ Faculty of Health, School of Health and Social Development Deakin University Geelong Australia

## Abstract

**Background:**

The Australian National Disability Insurance Scheme (NDIS) has transformed early intervention for children by introducing an individualised funding model. This PRISMA‐based scoping review explores the common characteristics of therapy providers and the barriers and facilitators to implementing child NDIS interventions.

**Method:**

Twenty‐seven databases were searched (2013–2026), and findings inductively grouped using qualitative content analysis. The methodological quality of included studies was assessed using the Mixed Methods Appraisal Tool.

**Results:**

Fifty‐five studies (*k*), representing a pooled sample of 3073 therapy providers, were reviewed. Qualitative designs were frequently used to understand therapists' experiences and perspectives (*k* = 37), followed by mixed methods (*k* = 12), quantitative designs (*k* = 5), and multiple methods (*k* = 1). Service barriers primarily stemmed from external structural factors, particularly NDIS policies and processes. Interventions tailored to a child's specific needs were a key facilitator.

**Conclusions:**

Whilst the NDIS is vital for child disability supports, therapy providers seek improvements to the scheme's complexity.

## Lay Summary

- This scoping review maps barriers and facilitators to intervention implementation in the Australian National Disability Insurance Scheme (NDIS), encompassing various therapy providers and childhood disabilities (e.g., neurodivergent, developmental and intellectual disabilities).
- Barriers were predominantly driven by external system‐level factors, with NDIS policies and administrative requirements being prominent obstacles to implementation.
- Common facilitators included interventions that could be tailored to each child.

## 1 Introduction

Research shows that early intervention and access to foundational services for children with cognitive disabilities, including neurodevelopmental disorders such as autism and intellectual disability, can significantly benefit their development and promote their independence as they move into adulthood (Kuld et al. 2023; Windsor et al. 2023). For these children, government schemes such as the Australian National Disability Insurance Scheme (NDIS) can fundamentally improve support access by providing funding for essential services, including assistance with daily living, education, employment, social engagement. Established in 2013, the NDIS replaced the National Disability Agreement, which involved multilateral arrangements between the federal government and its states and territories (Buckmaster and Clark 2018). This agreement utilised a block funding model, where providers were paid directly by the government to provide a specified amount of service to people with specified disabilities (Knight 2021). The previous system was described as ‘underfunded, unfair, fragmented, and inefficient’, because it failed to provide people with disabilities adequate choice, certainty of access to appropriate supports and opportunities for community participation (Productivity Commission 2011, 5). In comparison, NDIS eligibility requires a permanent disability or impairment that substantially reduces functional capacity for daily activities and social/economic participation (NDIS Act 2013 (Cth) s. 24). The NDIS represents a fundamental shift from the previous block funded model under the National Disability Agreement to an individualised model which gives eligible persons choice and control over their supports, enabling them to directly purchase needed services from providers (Disney et al. 2025).

However, there are challenges to implementing interventions within the NDIS—both for consumers who must navigate the system and find providers (Russo et al. 2021) and for providers who must adapt to a market characterised by increasing demand for a wider range of services (Foster et al. 2022; NDIS Review 2023). These providers include professions who develop participants' skills and independence, such as allied health (e.g., physiotherapy, occupational therapy, social work, psychology, speech pathology), nursing, developmental educators and behaviour therapists (Ding et al. 2025).

Given that children and adolescents (aged 0–19 years) with cognitive disabilities comprise almost 50% of NDIS participants (National Disability Insurance Agency [NDIA] 2023), research into the effective implementation of interventions that enhance their functional independence is crucial to both the scheme's success and participants' ongoing development and wellbeing. To date, available reviews of the NDIS have narrowly focused on: single disciplines (e.g., social work, music therapy; Bigby et al. 2018; Birch and Thompson 2023; Boaden et al. 2025); governance (D'Aprano et al. 2024; Jaques et al. 2018; Moran et al. 2024; O'Reilly et al. 2018); provider training (e.g., positive behaviour support; Jojo and Wilson 2024; Germeroth et al. 2023; Hall et al. 2025; Hayward et al. 2021); or provider experiences supporting adults with a disability (e.g., Lage et al. 2024; Lewis et al. 2017; Madsen et al. 2020). This review is the first, to the authors' knowledge, to comprehensively examine the experiences of therapists providing NDIS interventions for Australian children and adolescents with cognitive disabilities. The methodology of a scoping review was adopted given the diverse evidence base, particularly regarding how NDIS interventions are developed and designed (Munn et al. 2018). Our review was guided by the following research questions:

1. What are the characteristics of included studies, including research methods used (i.e., study aims, data analysis techniques, outcomes)?
2. What are the characteristics of therapy providers and child participants?
3. What are the common barriers and facilitators identified by therapy providers in implementing therapy interventions for this cohort?

## 2 Methods

### 2.1 Protocol and Registration

This scoping review is registered on the Open Science Framework (https://osf.io/s9n5b) and was guided by the JBI methodological framework (Peters et al. 2024) and complementary Preferred Reporting Items for Systematic reviews and Meta‐Analysis—Scoping Reviews (PRISMA‐ScR; Tricco et al. 2018; see Data S1). Additionally, included studies underwent critical appraisal to understand the quality of the available evidence.

### 2.2 Study Eligibility Criteria

The inclusion criteria, as outlined in Table 1, followed the Population, Concept and Context of interest (Peters et al. 2021). Both peer‐reviewed and grey literature (e.g., dissertations) were eligible, provided they were full‐texts with accessible primary data (including data accessible upon application to the corresponding author). Commentary, opinion articles, review articles and conference abstracts were excluded.

**TABLE 1:** Study eligibility criteria.

Population	Inclusion	Allied health professionals, nurses, or adjacent therapeutic service providers who deliver activities that are related to, or complementary to, core disability services funded by the NDIS^a^, including:		
		Allied Health Professions^b^	Art therapy	Orthoptics
			Audiology	Physiotherapy
			Counselling	Podiatry
			Dietetics	Psychology
			Exercise Physiology	Psychotherapy
			Music therapy	Social Work
			Occupational therapy	Speech pathology/therapy
		Adjacent Therapeutic Service Professions^c^	Behaviour therapy	Nursing
			Developmental educator	Personal Training
			Drama therapy	Play Therapy
			Early childhood professional	Therapy assistant
			Mentoring	
		Provide services funded by the NDIS to NDIS participants, and Provide therapeutic services, capacity building supports, or interventions to children and/or adolescents with cognitive disabilities (see Table S1 for list of included disabilities and search terms). ○ That is, any disability which impacts cognitive functioning, including both hereditary (e.g., autism, Fragile X syndrome) and acquired disabilities (e.g., traumatic brain injury). ○ Children and/or adolescents aged 0–19, as per the World Health Organisation's (WHO) definitions of childhood (WHO n.d.–b) and adolescence (WHO n.d.–a). ○ Heterogenous samples that include children, adolescents and adults may be included, provided that the outcomes are relevant to children with disabilities (e.g., the importance of effective communication between services providers/carers/family members).		
	Exclusion	Medical health professionals (e.g., General Practitioners, Paediatricians, etc.). Non‐allied health support workers or general disability support workers. Focus is on the support of adult participants. There is no reference to sample age or age range of clientele with disabilities (e.g., people with intellectual disabilities)		
Concept	Inclusion	Studies that investigate service barriers (i.e., factors that hinder, limit or prevent therapy provision) or service facilitators (i.e., factors that favour or facilitate therapy provision) for implementing interventions targeted towards children and/or adolescents with cognitive disabilities who are NDIS participants, including: ○ Intervention characteristics (e.g., design, composition of group members, etc.), ○ Systemic or environmental factors (e.g., availability of funding or parent capacity to support child/adolescent attendance). ○ Provider characteristics (e.g., self‐reported level of confidence or preparedness to support children/adolescent with cognitive disabilities), Barriers and/or facilitators to intervention implementation are identified by therapy providers themselves. For this review, therapy support will be broadly defined as any: ○ Named intervention (e.g., Sleeping Sound intervention) ○ Broad intervention (e.g., a Naturalistic Developmental Behavioural Intervention) ○ Ongoing therapy provision (e.g., case notes of fortnightly one‐hour sessions with an occupational therapist). ○ Exploration of therapist experiences providing support. The NDIS, by itself, may also be considered an intervention, provided that: ○ The NDIS is reported as a direct barrier or facilitator to supporting children or adolescents with cognitive disabilities. ○ The focus is on allied health, nursing, or adjacent/complimentary services for children and/or adolescents with cognitive disabilities.		
	Exclusion	Study explores the experiences of non‐NDIS service providers (e.g., parents, doctors). Study investigates supports that assist in the completion of activities rather than build capacity (e.g., support workers who assist with activities of daily living, such as dressing oneself, rather than assist participant to develop their independence in those skills).		
Context	Inclusion	Studies must make explicit reference to the NDIS in the body of the text, that is, ○ In the introduction, the NDIS is identified as a key context for the provision of disability supports in Australia, and/or ○ In the Methods, participants need to be identified as (or likely to be) NDIS participants, and/or ○ In the Results, some feature of the NDIS (as an organisation/system or funding source) must be identified as enabling or barring the implementation of an intervention, and/or ○ In the Discussion/Conclusion, practical implications of the findings for the NDIS are identified, and/or ○ Studies are funded (in part or in whole) by the NDIA or the NDIS Quality and Safeguards Commission.		
	Exclusion	Studies do not refer to the NDIS in one of the above inclusion criteria (e.g., a cursory reference to the NDIS in the References). Studies explore the views or experiences of Australian service providers outside the NDIS (e.g., service providers for aged care)		

^a^ Professions selected for this review were those eligible for NDIS funding (see https://ourguidelines.ndis.gov.au/media/1768/download?attachment and https://www.ndis.gov.au/media/7727/download?attachment). A list of excluded supports is available at: https://ourguidelines.ndis.gov.au/media/1769/download?attachment.

^b^ In Australia, Allied Health professions are regulated by the Australian Health Practitioner Regulation Agency (AHPRA) (see https://www.ahpa.com.au/allied‐health‐professions).

^c^ Adjacent therapeutic services refer to qualified professional services that are not registered with AHPRA.

### 2.3 Information Sources

Ten platforms, hosting 27 databases, were identified as appropriate information sources for this review (see Table 2).

**TABLE 2:** Included platforms and databases.

Clarivate	Current Contents
	Web of Science
Cochrane	Cochrane Online
EBSCOHost	Cinahl Ultimate
Elsevier	ScienceDirect
	Scopus
Informit	A+ Education
	Australian Policy Database
	Australian Public Affairs Full Text
	Informit
	Informit E‐Library: Health
	Multicultural Australia and Immigration Studies
OVID	Embase
	Emcare
	Medline
	PsycINFO
ProQuest	Coronavirus Research Database
	Eric (Online)
	Nursing and Allied Health Database
	Public Health Database
	ProQuest
	ProQuest Central
	Proquest Dissertations and Theses Global
PubMed	PubMed
	PubMed Online
Sage	Sage Journals
Wiley	Wiley Online Library

Platforms covered health and medical sciences, multidisciplinary, political, international and/or sociological research. Databases also needed to meet the following criteria:

- An index database:
- Covered the date range of the NDIS (i.e., 2013 to present);
- Had advanced search functionality, including Boolean operators and other advanced searching syntax.

### 2.4 Search Strategy

Advanced search strategies were used to create a more controlled query, by searching across different fields. Specifically, the ‘NDIS’ and ‘disability’ search strings, including MeSH terms where relevant, were searched in all fields to provide the widest search available to each platform. The ‘provider’ search terms were then limited to Title, Abstract and Keywords searches to ensure that allied health and adjacent or complimentary therapeutic services were the focus (see in Table S1 for search terms and Table S2 for an example logic grid and search strings). Where possible, date limits were set from 2013 onwards (corresponding with the onset of the NDIS). The database searches were conducted between March 17th, 2025 and March 25th, 2025. A subsequent search was conducted between March 24th and March 30th, 2026 to capture newly published studies and maintain the currency of the review.

Eligible records were exported to Zotero 7.0.15 by the first author, where they were sorted by database before exporting to Covidence Systematic Review Software (www.covidence.org) for automatic removal of duplicate records and further screening by two independent reviewers (MM and DD).

### 2.5 Data Extraction

Data from each included study were extracted by the first author (MM) and cross‐checked by the second (DD) using a purposely‐designed Microsoft Excel spreadsheet. Extracted data included: bibliographic information, methodological details (i.e., study aims, data collection, outcomes), therapy provider details (e.g., discipline, years of experience) and clientele data (disabilities and ages of child/adolescent samples).

The Mixed Methods Appraisal Tool (MMAT; Hong et al. 2018), which allows a comparative critical appraisal regardless of the study design, was used. A random subset of 10 studies (18%) was independently rated by the third and fourth authors (DT and AT), with 91% agreement reached. The few disagreements were resolved through consensus discussion.

### 2.6 Data Synthesis

Data were synthesised using a descriptive narrative approach, which is a form of textual summarisation. This approach involved numerical summaries of the bibliographic (i.e., publication date, author(s)) and quantitative data (i.e., participants, sample size, clientele) and deductive content analysis of the outcome data (Lockwood et al. 2019; Peters et al. 2024; Pollock et al. 2023). Data extraction and analysis was completed by the first author, who tabulated key findings in line with the aims of this scoping review.

The content analysis began with the development of an initial set of definitions for what constituted a ‘service barrier’ and a ‘service facilitator’ (see Table 1). Using these definitions as the coding framework, the first author read each study, extracted key data and outcomes and highlighted passages that aligned with these established definitions. Segments of text were further grouped into categories and subcategories as follows:

- Intervention characteristics such as the setting, intervention format and delivery, team composition and implementation processes.
- Systemic or environmental factors, including familial contexts, community resources or settings, service‐system structures and broader social and environmental conditions that influence intervention effectiveness and accessibility.
- Provider characteristics, or individuals' knowledge and capacity to deliver an intervention.

For qualitative studies, identified themes or codes (as listed in the original paper) were the basis for the categorisation. For quantitative studies, the numerical findings were the basis for the categorisation. To ensure accuracy, the second author (DD) double‐checked the analysis at the category and subcategory levels. Finally, the frequency (*n*) of each category and subcategory, based on how many unique barriers or facilitators were identified within each study, was reported.

### 2.7 Positionality Statement

The combined academic, clinical and professional perspectives within the team provided a foundation from which to analyse and discuss the findings, whilst also ensuring that the results had practical implications for future research on the NDIS. The first author, MM, brings a crucial person‐centred perspective to the analysis and discussion as a person with disability who has previously worked within the NDIS. The second author, DD, brings experience in neurorehabilitation practice and research, including an understanding of the systemic factors faced by the therapy providers examined in this study. DT is a senior academic with expertise in research methods and international partnerships which aim to improve support for people with disabilities. AT is a certified practising speech‐language pathologist with extensive experience in child disability services and inclusion research, informing their perspective on NDIS service challenges and broader disability services.

## 3 Results

### 3.1 Selection of Sources of Evidence

**FIGURE 1:**
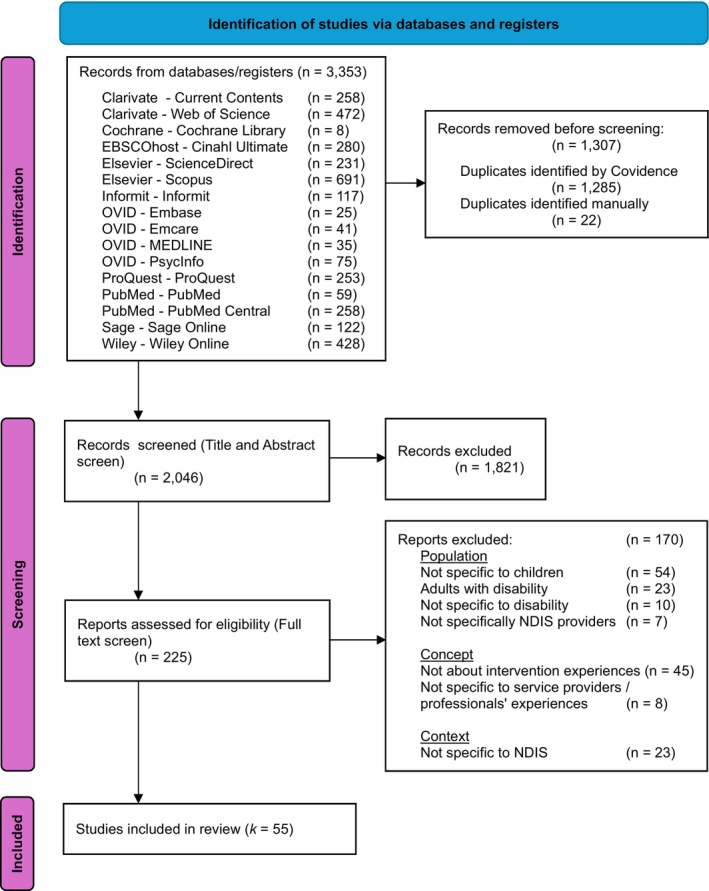
Flowchart of study selection adapted from PRISMA.

A total of 3353 potentially eligible studies were identified from the database searches (see Figure 1), with 2046 retained following the removal of duplicates. Inter‐rater reliability was moderate to good for each stage of the review (Title and Abstract proportionate agreement = 0.91, *κ* = 0.61; Full Text proportionate agreement = 0.93, *κ* = 0.81). The discrepancy in screening at the title and abstract stage reflected challenges in determining the sample eligibility criteria, with studies not routinely reporting mean age or age range of their child sample. To resolve this, both reviewers adopted a more inclusive definition whereby studies that included mixed child and adult groups but still reported outcome data relevant to children (e.g., providers' capacity to work with this cohort) were deemed eligible. This decision was consistent with scoping review methodology, which prioritises comprehensive mapping of the evidence base over restrictive exclusion criteria (Tricco et al. 2018). During screening, four studies with overlapping samples were additionally identified (Cashin, Pracilio, Buckley, Kersten, et al. 2022; Cashin, Pracilio, Buckley, Morphet, et al. 2022; Green et al. 2020, 2021). The final sample therefore comprised 55 studies (*k*), which contributed to 53 unique samples (see Table 3 for full citations and data extraction).

**TABLE 3:** Therapy providers listed in included studies.

Therapy providers	*k*	%
Occupational therapists	32	21.8
Speech pathologists/therapists	26	17.7
Psychologists	21	14.3
Physiotherapists	13	8.8
Nurses	12	8.2
Early childhood professionals/educators	8	5.4
Social workers	6	4.1
Applied behaviour analysis practitioners/behaviour therapists/behaviour support	5	3.4
Dietitians	4	2.7
Music therapists	3	2.0
Art therapists	2	1.4
Exercise physiologists	2	1.4
Allied health assistants	1	0.7
Developmental educators	1	0.7
Family therapists/practitioners	1	0.7
Genetic counsellors	1	0.7
Podiatrists	1	0.7
Allied health/therapists—not further specified	8	5.4

### 3.2 Study Characteristics

All 55 studies were sourced from peer reviewed journals, with most (*k* = 46; 85%) published from 2020 onwards. The majority of studies that met the inclusion criteria adopted qualitative study designs (*k* = 37; 67%; e.g., phenomenological, case studies, qualitative descriptive), with thematic analysis being the most common method employed (*k* = 23; 33%). Mixed methods (*k* = 12; 22%; e.g., concurrent convergent, multiple case study), quantitative (*k* = 5; 7%; e.g., cross‐sectional, observational studies) and multiple methods research (*k* = 1; 2%) were least common.

The included studies addressed various aims. The exploration and understanding of therapy providers' experiences, including how the health service's transition to the NDIS influenced their practice, was a key aim (*k* = 24; 44%). Additional primary aims included: the evaluation of a program, therapy tool, or intervention (*k* = 14; 25%; e.g., video‐conferencing for regional support); providers' perceptions or views on their practice (*k* = 8; 15%); perceived knowledge and/or preparedness for supporting clients with cognitive disabilities (*k* = 5; 9%); and improving or identifying barriers to service access (*k* = 4; 7%).

Interviews were the most common format of data collection (*k* = 31; 39%). Surveys (*k* = 25; 32%), focus groups (*k* = 12; 15%), reflective pieces (*k* = 2; 3%) and document reviews (*k* = 2; 3%; e.g., clinical records) were also utilised. Individual studies then adopted case studies (Grace et al. 2025), field notes (Jeremy et al. 2025), informal discussions and observations (Moir et al. 2022), online assessments (Young et al. 2023), process data (Hines et al. 2019) and workshops (D'Arcy et al. 2022).

Only four studies included validated measures as part of their intervention evaluation. Britt et al. (2025) captured service providers' beliefs about family centred practices—including its philosophy, principles, outcomes and personal barriers (Measures of Beliefs about Participation in Family‐Centred Service; King et al. 2003). This same study also examined providers' engagement in family‐centred care (Measures of Processes of Care—Service Providers; Britt et al. 2025; Woodside et al. 2001). Hines et al. (2019) used the Canadian Occupational Performance Measure to assess self‐perceived performance in everyday activities (Law et al. 1990). Paton and Hiscock (2019) interviewed health professionals about the components of optimal primary paediatric care based on the Models of Child Health Appraised project (Brenner et al. 2017). Finally, Young et al. (2023) utilised the Institute of Work Psychology Multi‐Affect Indicator (Warr 1990) to measure job related wellbeing among key workers and their managers as well as perceptions of supervisory support (Perceived Supervisor Support scale; Eisenberger et al. 1986).

Two additional studies used customised measures based on Bandura's (2006) self‐efficacy theory to evaluate workers' or managers' self‐confidence (Young et al. 2023) and an adapted Supports and Barriers Questionnaire (Rivard et al. 2010) to capture providers' views on implementing routine clinical assessments in disability services (Kerr et al. 2023).

### 3.3 Sample Characteristics

The pooled sample included 3073 therapy providers (*M*: 57.98, SD: 115.03, range: 1–693), with years of work experience ranging from 0 to 60 (*M*: 14.19 years, SD: 6.88, *k* = 8). Most studies targeted multiple disciplines as their study participants (*k* = 35; 64%), including occupational therapists (*k* = 32; 21.8%), speech therapists (*k* = 26; 17.7%), psychologists (*k* = 21; 14.3%), physiotherapists (*k* = 13; 8.8%), nurses (*k* = 12; 8.2%) and early childhood professionals (*k* = 8; 5.4%). Seven studies described their sample as ‘Allied Health’ (Barr et al. 2024; Green et al. 2020; Green et al. 2021; Mozolic‐Staunton et al. 2021; Vlcek et al. 2020) or ‘Key Therapist’ (Luskin‐Saxby et al. 2023) (see Table 3 for full list).

Forty‐two different types of disabilities were represented among the included studies, 17 of which were of interest to this review (Table 4). Neurodivergence (e.g., autism) was well represented, as were developmental disabilities and intellectual disabilities. Mixed child and adolescent caseloads were common (*k* = 24; 45%), as were caseloads comprised solely of children (0–12 years of age; *k* = 18, 34%). Three studies incorporated a mixed cohort of children, adolescent and/or adult participants in their caseloads (Hill et al. 2023; Son et al. 2019; Whitehead et al. 2022).

**TABLE 4:** List of included disabilities that service providers supported.

Disabilities of interest	*k*	%
Neurodivergence	46	42.2
Autism	35	32.1
Attention deficit disorder (with or without hyperactivity)	4	3.7
Neurodevelopmental conditions (not further specified)	7	6.4
Behavioural and attentional difficulties (not further specified)	2	1.8
Developmental disabilities/disorders	21	19.3
Developmental delay	11	10.1
Global developmental delay	3	2.8
Developmental language disorder	2	1.8
Developmental disabilities (not further specified)	5	4.6
Intellectual disabilities	23	21.1
Intellectual disability	19	17.4
Learning disability/difficulties	4	3.7
Intellectual conditions (not further specified)	1	0.9
Speech, language and communication disorders	5	4.6
Speech, language and communication disorder/disability (not further specified)	4	3.7
Speech and/or language difficulties/delays	1	0.9
Speech sound disorder	1	0.9
Foetal alcohol spectrum disorder	4	3.7
Brain injury	3	2.8
Genetic/chromosomal disorders	3	2.8

*Note:* *k* = number of studies providing these data.

### 3.4 Critical Appraisal

All included studies met the MMAT filtering questions, indicating that they were appropriate for further quality assessment (Hong et al. 2018; see Table S4 for quality appraisal results).

The 37 qualitative studies employed suitable designs and data‐collection methods, and their interpretations were well supported by the data and accompanying quotes. Coherence across data sources, analysis and interpretation was generally strong, although Green et al.'s (2021) drew concern for shifting from an inductive thematic analysis to a deductive framework after generating categories.

One third of the 12 mixed‐methods studies failed to justify their use of this design, describing procedures rather than providing a rationale what they did. Nonetheless, all studies integrated and interpreted their qualitative and quantitative components based on the specific quality standards of their methodology traditions. Most also addressed any discrepancies between qualitative and quantitative findings, with the exception of Evans et al. (2024).

All four descriptive quantitative studies employed relevant and representative sampling. However, both studies undertaken by Cashin, Pracilio, Buckley, Kersten, et al. (2022) and Cashin, Pracilio, Buckley, Morphet, et al. (2022) relied on snowball sampling and did not report response rates, making non‐response bias difficult to judge. Aside from this, measurement tools and statistical analyses were appropriate to answering the research questions across studies.

Only Young et al. (2023) used a non‐randomised quantitative design. Its sample was representative of the target population, measures were appropriate and confounders were adequately addressed in the analysis. However, outcome data were incomplete—attrition was 13%, driven by low survey completion among parents (76%), with therapy provider attrition unreported. Further, confounders were not explicitly discussed in their design and analyses, making it difficult to tell if they were sufficiently accounted for.

Kefford et al. (2025) used a well‐aligned multiple methods design. The qualitative component was appropriate for their research question and data collection approach. The findings and interpretation of results were also supported by the data, demonstrating coherence across data collection, analysis and interpretation. The quantitative analysis followed a descriptive approach to a relevant and representative sample. The measurements and analyses were also appropriate to address the research question. However, the quantitative findings were limited by significant non‐response bias, with a third of the surveys excluded due to incomplete data.

### 3.5 Barriers to NDIS Service Implementation

Across the 55 studies, 18 codes related to implementation barriers were identified, representing 171 individual barriers extracted from the studies (see Table 5).

**TABLE 5:** Identified service barriers grouped by categories and subcategories.

Category (*n*)	Subcategory (*n*)
Intervention characteristics (30)	Issues with intervention delivery/design (15)
	Difficulties within provider/team composition (12)
	Individual child difficulties (3)
Systemic/environmental factors (125)	Complex NDIS policies and processes (33)
	Difficulties arising from family circumstances/capacity/concerns/beliefs (28)
	Inadequacies in the services and/or the service environment (21)
	Organisational limitations (resources, practices and processes) (15)
	Limited availability of services (7)
	Inadequate oversight (6)
	Inadequate training (4)
	Complex socio‐political/historical contexts (3)
	Service disruptions (COVID, etc.) (3)
	Limited access to resources and evidence (2)
	Difficulties adapting therapy/intervention to specific contexts (1)
	Ambiguous roles/expectations/responsibilities (1)
	Service location difficulties (1)
Provider characteristics (16)	Lack of experience and Inadequate training (15)
	Clinician's personal biases (1)

*Note:* *n* = number of barriers identified under each category and subcategory.

Intervention barriers primarily revolved around delivery issues, such as limited session time and fidelity challenges. Additional difficulties included provider/team composition conflicts (e.g., differing agendas/values) and challenges adapting interventions to client needs (e.g., children not engaging with telehealth).

Systemic or environmental barriers focused on the NDIS itself, including how the policies and processes have had a transformative impact on both service providers and the broader Australian disability landscape. Family circumstances (e.g., beliefs, capacity), service access issues (e.g., difficulties navigating a complex service environment) and poor organisational resources, practices and processes (e.g., limited professional development and training opportunities, high caseload) were also cited. Less common barriers included: service unavailability (e.g., waiting periods, costs); poor clinical oversight (including lack of governing bodies or best practice guidelines); inadequate training (e.g., undergraduate training not adequately preparing clinicians to assess or support disability populations); complex socio‐political/historical contexts (e.g., Indigenous Australians' trust of health organisations); service disruptions (e.g., Covid); difficulties adapting therapy into a school's curriculum framework; role ambiguity (e.g., broader debate about responsibility of assessing disabilities, such as Foetal Alcohol Spectrum Disorder); and geographic remoteness hindering the effective delivery of interventions.

Provider‐related barriers mainly stemmed from limited experience and training, including insufficient disability‐specific knowledge and feeling ill‐equipped to address disability—broadly or specifically. Personal biases and views were also raised by Kerimofski et al. (2025) as impeding effective practice.

### 3.6 Facilitators to NDIS Service Implementation

Across the included studies, 122 different service facilitators were identified, grouped into 14 different subcategories (see Table 6).

**TABLE 6:** Identified service facilitators (*n*) grouped by categories and subcategories.

Category (*n*)	Subcategory (*n*)
Intervention characteristics (51)	Flexibility of approach/personalised support or service (25)
	Appropriate intervention delivery (14)
	Good provider/team composition and collaboration (12)
Systemic/environmental factors (30)	Adaptability to different service delivery contexts (8)
	Supportive organisational structures, resources, relationships and practices (8)
	Family‐centred collaboration and communication (5)
	Benefits of service location (4)
	Support access due to NDIS funding (2)
	Access to outside supports (2)
	Preexisting networks and connections (1)
Provider characteristics (41)	Positive approaches to therapeutic practice (16)
	Personal development of knowledge and understanding (14)
	Clinician's personal experiences/motivations (9)
	Existing individual resources (2)

*Note:* *n* = number of barriers identified under each category and subcategory.

Flexibility of approach and personalisation were perceived as key factors for success, enabling adaptation of interventions, better child engagement and positive child outcomes. Other important facilitators included using validated assessment tools to accurately measure progress, setting scaffolded goals and ensuring effective therapy team composition with clear interprofessional communication and role statements.

Key systemic or environmental facilitators included supportive policies that encouraged parental involvement, strong organisational resources (e.g., professional development opportunities, transdisciplinary service) and positive family circumstances (e.g., informed parents enabling care). Less common facilitators included service location (e.g., remote or rural community ties and co‐location with other services), the impact of NDIS funding, access to specialised child supports (e.g., nurse navigators, autism advisors) and the existence of or connection to pre‐existing professional networks and connections.

Provider characteristics were considered crucial, particularly a therapist's ability to develop relationships with families and encouraging their participation. Related facilitators included an individual's knowledge and understanding, their skills/personal characteristics (e.g., enthusiasm and values) and access to reliable resources for intervention decisions (i.e., websites, books, podcasts, scholarly articles).

## 4 Discussion

This scoping review is the first to identify and summarise the literature regarding provider‐identified barriers and facilitators to the implementation of therapeutic interventions for children and adolescents with cognitive disabilities in the context of the NDIS. Our findings from 55 studies highlight the complex experiences and perspectives of individual therapy providers. The realities of navigating the NDIS were readily identified alongside factors that facilitated intervention success, including the characteristics of both therapy providers and participants themselves.

The most common professions represented in NDIS therapeutic support research (i.e., occupational therapists, speech pathologists and psychologists) aligns with the NDIS funding model and the types of services most frequently utilised by participants (Pye et al. 2024). These allied health professions are key providers of NDIS capacity‐building support, helping participants develop crucial life skills to enhance their independence and improve their ability to participate in community (Ding et al. 2025; NDIA 2024b, 2025b). Ideally, datasets from the NDIS should also detail the types of providers that participants use, including the number of hours spent and the types of therapy work they undertake, to ascertain if the distribution of providers in the included studies is representative of therapy support usage within the scheme more broadly.

Therapy providers identified more barriers (*n* = 170), particularly those related to the NDIS itself, than facilitators (*n* = 124); although the minor difference suggests a potential negativity bias (Rozin and Royzman 2001). Given the considerable role that the NDIS plays in the Australian disability landscape and its focus in this review, it is not surprising that the NDIS itself was identified as a significant barrier. The advent of the NDIS has significantly changed, and often increased, administrative workloads due to more complex quality requirements, greater detail needed in reports and the need to record billable hours (Campbell et al. 2025; Dintino et al. 2019; Gavidia‐Payne et al. 2024; Kefford et al. 2025; Kerr et al. 2023; Zagler et al. 2024). Increased reporting requirements have reduced therapy providers' time for clinical work and family support, particularly in disadvantaged communities where providers have had to become advocates and social workers to help families navigate the NDIS system and understand decisions made by NDIA. These increasing demands may also contribute to a current landscape that discourages interdisciplinary collaboration. This disconnect affects both cooperation among disability providers supporting the same participant and integration with mainstream health and mental health services (Leif et al. 2020; Paton and Hiscock 2019; Philpott‐Robinson et al. 2024; Son et al. 2019; Whitehead et al. 2022). Moreover, an increased workload can lead therapists to using a reactive, crisis‐management approach—an approach which is inconsistent with NDIS principles which emphasise strategies to build capacity to undertake activities independently (NDIS Act 2013 (Cth) s. 4.11).

Access to the NDIS scheme or appropriate supports was further hindered by complex navigation of the system and inadequate support for transitioning to the scheme, particularly for marginalised groups (e.g., children of migrant parents or Aboriginal Australian peoples; Boaden et al. 2021; Dintino et al. 2019; Edwards et al. 2025; Gavidia‐Payne et al. 2024; Green et al. 2021; Lee et al. 2018; Leif et al. 2020; McInerney et al. 2020; Mozolic‐Staunton et al. 2021; Newton et al. 2025; Nickless et al. 2023; Philpott‐Robinson et al. 2024). These systemic barriers echo those captured in the NDIS Review (2023) recent *Working Together* report.

In combination, these findings highlight the importance of parents being provided clear and accessible information about evidence‐based therapies within the NDIS so that they can make informed decisions. Indeed, included studies commented that parents were burdened by finding suitable services (Gavidia‐Payne et al. 2024; Hill et al. 2023; Nickless et al. 2023; Paton and Hiscock 2019; Son et al. 2019; Trembath et al. 2016), hampered by a lack of information and system support (Chan et al. 2023; Gaffney et al. 2024; Graham et al. 2025; Hsiang et al. 2024; Leong et al. 2025; Mills et al. 2023; Newton et al. 2025) and competing family demands—especially when caring for children with complex medical needs, or when families faced socioeconomic or geographical disadvantage (Chan et al. 2023; Gaffney et al. 2024; Hill et al. 2023; Johnsson and Bulkeley 2021; Mills et al. 2023; Philpott‐Robinson et al. 2024). The NDIS, through its website and partner program, could serve as invaluable resources for such guidance, coupled with a need to include and communicate with families throughout the intervention process (Gaffney et al. 2024; Green et al. 2021; Jeremy et al. 2025; Johnsson et al. 2019; Leong et al. 2025; Paton and Hiscock 2019; Philpott‐Robinson et al. 2024; Son et al. 2019). Efforts are, however, needed to prevent the NDIS from entrenching existing inequities (Boaden et al. 2021; Malbon et al. 2019). Recent efforts to increase the participation of people with disabilities in the NDIS' design and administration (e.g., co‐design working groups, feedback sessions) in addition to NDIS' early childhood approach (NDIA 2024a), which reinforces parent or caregiver participation and engagement in child‐focused interventions (Chan et al. 2023; Gaffney et al. 2024), are important first steps. Ideally these approaches should be coupled with direct funding to support families to access and coordinate services.

Service facilitators were identified to overcome these challenges, including the need for therapy providers to adapt and personalise their interventions to suit individual clients' circumstances, goals and preferred communication style. Whilst this tailored approach is consistent with person‐centred and disability affirming practice (Barr et al. 2024; Grace et al. 2025; Hines et al. 2019; Pinney 2022), it also requires flexibility and creativity to use a variety of approaches, in various settings, to respond to each client's unique needs in the moment (Arns and Thompson 2019; Graham et al. 2025; Hsiang et al. 2024; Kefford et al. 2025; Moir et al. 2022; Newton et al. 2025; Sulek et al. 2024; Whitehead et al. 2022). To facilitate this, providers must stay up‐to‐date with the evidence and research base within their profession. Importantly, the NDIS requires that licensed therapists maintain their skills and stay current with best practice through ongoing professional development and adherence to their organisational body's qualification standards (NDIS Quality and Safeguards Commission 2025). In this way, providers can sustain the overall goal of building participant capacity.

### 4.1 Limitations of the Scoping Review and Implications for Future Research

Several limitations may impact the overall findings of the present review. Despite this review aiming to include a broad range of providers, occupational therapists, speech therapists and psychologists were overrepresented. Without clearer data from the NDIA as to what service providers are used, by whom, for what reasons, and in what quantities, it is not possible to determine if these numbers reflect their actual usage. The NDIA should consider collecting and providing this data, as this may provide clarity to researchers as to how best to orient their research, ultimately aiding in the long‐term sustainability of the scheme.

Another significant limitation is the underrepresentation of adolescents with cognitive disabilities in the literature, with only 27 studies including adolescents alongside other age groups, and a mere two focusing exclusively on this population (Son et al. 2019; Whitehead et al. 2022). As we have previously reported, NDIS research exploring adolescents (aged 13–19) with disabilities, particularly older adolescents, remains a critical, ongoing need (Marveggio et al. 2025).

Studies were skewed towards neurodivergent conditions, particularly autism. Although this focus aligns with the overall disability prevalence data for people under 65 years of age in Australia (Australian Bureau of Statistics 2024), dedicated research for other childhood disabilities (e.g., Fragile X syndrome) could reveal barriers faced by low incidence disability groups, potentially enhancing NDIS supports and interventions.

Future research should also explore the impact of the Australian Government's recent Thriving Kids initiative (NDIA 2025a), announced during the conduct of this scoping review. Thriving Kids is an early intervention pathway for children aged 8 years and younger with developmental delays or autism and low‐to‐moderate support needs (NDIA 2026). Set to roll out from October 2026 to January 2028, the program will replace individualised NDIS funding packages for these children with Foundational Supports. Although specific service details are yet to be released, Federal, State and Territory governments have agreed on a broad model that focuses on: boosting community and family awareness of developmental differences; expanding routine childhood health assessments for better early identification; and providing evidence‐based parenting programs. Additionally, it will offer targeted capacity‐building supports—ranging from individual and group sessions delivered by allied health professionals to more intensive programs (Department of Health, Disability and Ageing 2026).

However, this transition potentially risks reducing the intensity and specificity of the therapy supports currently accessible to these participants. It remains to be determined whether these new Foundational Supports can match or exceed the developmental gains previously achieved under intensive, NDIS‐funded early intervention models (Marveggio et al. 2025). Consequently, future research should compare the functional outcomes of children accessing Thriving Kids against those remaining on the NDIS, outlining the constraints and advantages of both pathways. This line of inquiry will help ensure that all children with cognitive disabilities benefit from systems that facilitate seamless, equitable access to proven early intervention.

The reliance on qualitative data among the included studies is expected, considering that our review question lends itself to qualitative methodologies. Whilst these data provide needed depth and context to the current evidence base, this also meant there was less focus on the use of validated measures to operationalise service barriers and facilitators. The generalisability and objective reliability of our findings could be strengthened in future qualitative work by adopting methods such as triangulation (using multiple data sources) or by integrating qualitative and quantitative evidence for a more comprehensive understanding, given both the paucity of either mixed methods or quantitative research.

Given that providers indicated a lack of experience and disability‐specific knowledge as significant barriers, an exploration of the curricula of university and other accredited tertiary programs might also be warranted. This audit should assess the inclusion of dedicated disability modules and practical placements within these programs to ensure future therapists are well‐prepared to provide services to individuals with disabilities. Entities such as the NDIA or governing bodies such as AHPRA might then be best positioned to design and deliver these professional development programs, thereby ensuring that appropriate and up‐to‐date disability training is being delivered, aligned with best practices. Such training could provide the NDIA with an opportunity to clarify their reporting requirements, so that therapists accurately identify and communicate the disability needs of their clients back to the NDIA.

## 5 Conclusion

This scoping review mapped study characteristics, therapy provider and child participant profiles and the key factors shaping service delivery within the context of the NDIS. Qualitative studies were most common, as was a focus on autism and child (aged 0–12) participants. Systemic barriers—particularly those linked to NDIS policies and processes—were most frequently reported, while tailoring interventions to individual child needs consistently emerged as a facilitator. Our findings highlight the value of the NDIS, while underscoring providers' calls to reduce scheme complexity to support effective therapy provision. Continued research and collaboration with therapy providers are essential to ensure best practices are delivered.

## Funding

MM and this research was supported by the Commonwealth through an Australian Government Research Training Program Scholarship (DOI: https://doi.org/10.82133/C42F‐K220).

## Ethics Statement

The authors have nothing to report.

## Supporting information


**Data S1:** Preferred Reporting Items for Systematic reviews and Meta‐Analyses extension for Scoping Reviews (PRISMA‐ScR) Checklist.

**Table S1:** Key concepts and search terms.

**Table S2:** Example of search terms (for Scopus) in concept search strings and connecting Boolean operator.

**Table S3:** Study Extraction.

**Table S4:** Mixed Methods Appraisal Tool.

## Data Availability

The data that support the findings of this study are openly available in Open Science Framework at https://osf.io/s9n5b/overview?view_only=e1c58d1328ba4c139d2ab4b4e75c6b9a.
