# Supplementary material for: A Scoping Review of Therapy Provision Under the National Disability Insurance Scheme: Provider‐Identified Barriers and Facilitators
Source: J Appl Res Intellect Disabil. 2026 Jul 10;39(4):e70278. doi: 10.1111/jar.70278 (PMC13354713; doi:10.1111/jar.70278)
Supplement: Supplementary file 2 — Table S1: Key concepts and search terms. [file JAR-39-e70278-s005.docx]

**Search Terms**

**Key Concepts**

| Concept 1 | Concept 2 | Concept 3 |
| --- | --- | --- |
| Cognitive Disability / Impairment | The NDIS | Service Providers |

**Keywords, Synonyms, and Search Strings – Concept 1**

| **Cognitive Disability / Impairment** | | | | |
| --- | --- | --- | --- | --- |
| **Keywords** | | **Synonyms** | **Truncations and wildcards (PsycInfo, Embase, Scopus (Trunc only))** | **MeSH Terms + PubMed Keywords**  **(Use both UK/US)** |
| Cognitive Disability | | Cognitive Disability | Cognitive Disab* | “Cognitive Dysfunction"[Mh] OR  Synonyms*[tiab] |
|  |  | Cognitive Impairment | Cognitive Impair* |  |
| Becomes | (Cognitive ADJ1 (Disab* OR Impair*)) | | | “Cognitive Dysfunction"[Mh]  OR Cognitive Disability[tiab] OR  Cognitive Impairment[tiab] |

| Intellectual Disability | Intellectual Disability | Intellectual Disabilit* | “Intellectual Disability”[mh] OR  Synonyms[tiab] |
| --- | --- | --- | --- |
|  | Intellectual Impairment | Intellectual Impairmen* |  |
| Becomes | (Intellectual ADJ1 (Disabilit* OR Impairmen*)) | | “Intellectual Disability”[mh] OR  Intellectual Disability[tiab] OR  Intellectual Impairment[tiab] |

| Autism Spectrum Disorder | Autism Spectrum Disorder | Autis* | "Autism Spectrum Disorder"[mh] OR  Synonyms [tiab] |
| --- | --- | --- | --- |
|  |  |  | "Autism Spectrum Disorder"[mh] OR Autism[tiab] OR Autistic[tiab] OR Asperger Syndrome[tiab] OR Aspergers Syndrome[tiab] OR Asperger’s Syndrome[tiab] |
|  | Asperger Syndrome | Asperge* |  |
| Becomes | Autis* OR Asperge* | |  |

| **Keywords** | **Synonyms** | | **MeSH Terms + PubMed Keywords**  **(Use both UK/US)** |
| --- | --- | --- | --- |
| Congenital Conditions | F?etal Alcohol Spectrum Disorder | Fetal Alcohol Spectrum Disorders[mh] OR Synonyms | |
|  | F?etal Alcohol Disorder |  |  |
| Becomes | F?etal Alcohol Spectrum Disorder OR F?etal Alcohol Disorder OR F?etal Alcohol Syndrome | Fetal Alcohol Spectrum Disorders[mh] OR Fetal Alcohol Spectrum Disorder[tiab] OR Foetal Alcohol Spectrum Disorder[tiab] OR Fetal Alcohol Disorder[tiab] OR Foetal Alcohol Spectrum Disorder[tiab] | |
| Other Congenital Conditions | Microcephaly | Microcephaly OR  Fetal hydantoin syndrome [Supplementary Concept] | |
|  | F?etal hydantoin syndrome |  |  |
| Becomes | Microcephaly OR F?etal hydantoin syndrome | Microcephaly[mh] OR Microcephaly[tiab] OR  Fetal hydantoin syndrome[tiab] OR Foetal hydantoin syndrome[tiab] | |

| Down Syndrome | Down Syndrome | “Down Syndrome”[mh] OR Synonyms |
| --- | --- | --- |
|  | Downs Syndrome |  |
|  | Down’s Syndrome |  |
| Becomes | ((Down OR Down’s OR Downs) ADJ1 Syndrome) | Down Syndrome[mh] OR Down Syndrome[tiab] OR Down’s Syndrome[tiab] OR Downs Syndrome[tiab] |

| ADHD | Attention Deficit Hyperactivity Disorder | “Attention Deficit Disorder with Hyperactivity”[mh] |
| --- | --- | --- |
|  | Attention Deficit Disorder |  |
| Becomes | Attention Deficit Hyperactivity Disorder OR Attention Deficit Disorder | Attention Deficit Disorder with Hyperactivity[mh] OR Attention Deficit Hyperactivity Disorder[tiab] OR Attention Deficit Disorder[tiab] |

| Processing Disorders | Language Disorder | “Language Development Disorders”[mh] AND synonyms |
| --- | --- | --- |
|  | Specific Language Disorder |  |
|  | Developmental Language Disorder |  |
|  | Speech Language Disorder |  |

| **Keywords** | **Synonyms** | **MeSH Terms + PubMed Keywords**  **(Use both UK/US)** |
| --- | --- | --- |
| Becomes | Language Disorder? | Language Development Disorders[mh] OR Language Disorder[tiab] |
| Processing Disorders | Sensory Processing Disorder | Auditory Perceptual Disorders[mh] AND/OR Synonyms |
| Becomes | Sensory Processing Disorder? | Auditory Perceptual Disorders[mh] AND Auditory Perceptual Disorders[tiab] |

| Developmental Delays | Global Developmental Delay |  |
| --- | --- | --- |
|  | Developmental Delay | Developmental Disabilities |
|  | Pervasive Developmental Disorder |  |
|  | Developmental NEAR/0 (delay OR disability OR disorder) | Child Development Disorders, Pervasive (covers ASD and associated concepts/terms) |
|  | Learning NEAR/0 (Disorder OR Disability) | Learning Disabilities |
|  | Neurodevelopmental Disorder | Neurodevelopmental Disorders (very broad, covers many of the above listed, such as ASD, ADHD, etc) |
| Becomes | (Developmental NEAR/0 (delay OR disability OR disorder)) OR  Learning Disorder OR  Neurodevelopmental Disorder | Developmental Disabilities[mh] OR  Developmental Disabilities[tiab] OR Developmental Delay[tiab] OR  Learning Disabilities[mh] OR Learning Disorder[tiab] OR  Child Development Disorders, Pervasive[mh] OR Pervasive Developmental Disorder[tiab] OR Neurodevelopmental Disorder[tiab] |

**Keywords, Synonyms, and Search Strings – Concept 2**

| **The NDIS** | |
| --- | --- |
| **Keywords** | **Synonyms** |
| National Disability Insurance Scheme | National Disability Insurance Scheme |
|  | National Disability Insurance Agency |

NDIS as author keyword

PsycINFO and potentially others that allow Author Keyword only

Key concepts – author keyword

**Keywords, Synonyms, and Search Strings – Concept 3**

| **Service Providers** | | |
| --- | --- | --- |
| **Keywords** | **Synonyms** | **MeSH Terms + PubMed Keywords**  **(Use both UK/US)** |
| Therapists | Occupational Therapist | Occupational Therapists OR Occupational Therapy |
|  | Occupational Therapy OR Occupational Therapist Occupational therapies OR Ergotherapy OR Ergotherapies |  |
|  | Speech Therapist | Speech-Language Pathology OR Speech Therapy |
|  | Speech Therapist OR Speech Therapy OR Speech Therapies OR  Speech patholog* |  |
|  | Art Therapist | Art Therapy |
|  | Art Therapist OR Art Therapy OR Art Therapies |  |
|  | Music Therapist | Music Therapy |
|  | Drama Therapist | Psychodrama |
|  | Drama Therapy OR Drama Therapist OR Dramatherapy |  |
|  | Behaviour Therapist |  |
|  | Play Therapist OR Play Therapy OR Play Therapies | Play therapy |
|  | Play Therapist OR Play Therapy OR Play Therapies |  |
| Becomes | Therap* OR  Ergotherap* OR  Dramatherap* OR  Speech patholog* | (Occupational Therapists OR Occupational Therapy OR Speech-Language Pathology OR Speech Therapy OR Art Therapy OR Music Therapy OR Psychodrama OR Play therapy).mh. |

| **Keywords** | **Synonyms** | **MeSH Terms + PubMed Keywords**  **(Use both UK/US)** |
| --- | --- | --- |
| Counsellors | Counsellor | Counseling OR Counselors |
|  | Rehabilitation Counsellor |  |
| Becomes | Counselling OR Counseling OR Counsellor OR Counselor | (Counseling OR Counselors).mh. |

| Exercise and Health | Physiotherapist | Physical Therapists |
| --- | --- | --- |
|  | Physiotherapist OR Physical Therapy OR Physiotherapy |  |
|  | Exercise Physiologist | Physical Therapy Modalities |
|  | Exercise Physiologist OR Exercise Physiology |  |
|  | Personal Trainer | Personal Trainers OR Athletic Trainers |
|  | Personal Trainer OR Athletic Trainer OR Fitness Coach or Fitness Trainer |  |
|  | Dietitian | Dietetics OR Nutritionists |
|  | Dietitian OR Dietetics |  |
|  | Podiatrist | Podiatry |
|  | Podiatrist OR Podiatry OR Chiropody |  |
| Becomes | Physiotherap* OR (Physical NEAR/0 Therap*) OR (Exercise NEAR/0 Physiolog*) OR ((Personal OR Athletic OR Fitness) NEAR/0 (Traine* OR Coach)) OR Dietitian OR Dietetics OR Podiatr* OR Chiropody | Physical Therapists OR Physical Therapy Modalities OR Personal Trainers OR Athletic Trainers OR Dietetics OR Nutritionists OR Podiatry |

| Health, Social, and Developmental | Social Worker | Social Workers OR Social Work |
| --- | --- | --- |
|  | Social Work OR Social Worker |  |
|  | Nurse | Nurses OR Nursing |
|  | Nurse OR Nursing |  |
|  | Psychologist | Psychologists OR Psychology |
|  | Psychologist OR Psychology OR Psychological Therapy OR Psychotherapy |  |
|  | Developmental Educator |  |
|  |  |  |
| **Keywords** | **Synonyms** | **MeSH Terms + PubMed Keywords**  **(Use both UK/US)** |
| Becomes | (Social NEAR/0 Wor*) OR Nurs* OR Psycholog* OR (Psycholog* NEAR/0 Therap*) OR (Developmental NEAR/0 Educator) OR Psychotherap* | Social Workers OR Social Work OR Nurses OR Nursing OR Psychologists OR Psychology |

| Other Professionals | Early childhood professional |  |
| --- | --- | --- |
|  | Audiologist | Audiology OR Audiologists |
|  | Audiology OR Audiologist |  |
|  | Orthoptist | Orthoptics |
|  | Orthoptist OR Orthoptics |  |
|  | Therapy assistant | Physical Therapist Assistants |
|  | Therapy assistant OR Therapy assistants |  |
| Becomes | (“Early childhood” NEAR/0 profession*) OR Audiolog* OR Orthopt* OR (Therapy NEAR/0 assistant*) | Audiology OR Audiologists OR Orthoptics OR Physical Therapist Assistants |

| Mentors | Mentor | Mentors |
| --- | --- | --- |
|  | Peer Mentor |  |
| Becomes | Mentor* | Mentors |
