# Supplementary material for: A Scoping Review of Therapy Provision Under the National Disability Insurance Scheme: Provider‐Identified Barriers and Facilitators
Source: J Appl Res Intellect Disabil. 2026 Jul 10;39(4):e70278. doi: 10.1111/jar.70278 (PMC13354713; doi:10.1111/jar.70278)
Supplement: Supplementary file 3 — Table S2: Example of search terms (for Scopus) in concept search strings and connecting Boolean operator. [file JAR-39-e70278-s002.docx]

| Supplementary Table 2. Example of search terms (for Scopus) in concept search strings and connecting Boolean operator. | | | |
| --- | --- | --- | --- |
| Search Line 1:  Disability String | (((Cognitive PRE/2 Disab*) OR (Cognitive PRE/2 Impair*)) OR ((Intellectual PRE/2 Disab*) OR (Intellectual PRE/2 Impair*)) OR (Autis* OR Asperge*) OR ({Fetal Alcohol Spectrum Disorder} OR {Fetal Alcohol Disorder} OR {Fetal Alcohol Syndrome} OR {Fetal hydantoin syndrome}) OR ({Foetal Alcohol Spectrum Disorder} OR {Foetal Alcohol Disorder} OR {Foetal Alcohol Syndrome} OR {Foetal hydantoin syndrome}) OR (“Microcephaly”) OR ("Down Syndrome" OR "Down’s Syndrome" OR "Downs Syndrome") OR (Language PRE/0 Disorder*) OR ({Sensory Processing Disorder} OR {Sensory Modulation Disorder}) OR ((Developmental PRE/0 delay) OR (Developmental PRE/0 disab*) OR (Developmental PRE/0 disorder*)) OR ({Attention Deficit Hyperactivity Disorder} OR {Attention Deficit Disorder}) OR ((Learning PRE/0 Disorder*) OR (Learning PRE/0 Disab*)) OR ((Neurodevelopmental PRE/0 disab*) OR (Neurodevelopmental PRE/0 disorder*)) OR (“PDD-NOS” OR “PDDNOS”) OR (“Angelman” OR “Coffin-Lowry” OR “Coffin-Siris” OR “Cornelia de Lange” OR “Cri du Chat” OR “Fragile X” OR “Kabuki” OR “Patau” OR “Trisomy 13” OR “Prader-Willi” OR “Rett” OR “Hurler” OR “San Fillipo” OR “Sanfilippo” OR “San Filippo” OR “Scheie” OR “Hurler-Scheie” OR “Hunter” OR “Morquio” OR “Maroteaux-Lamy” OR “Sly”) OR (“Gaucher” OR “Niemann-Pick” OR “Sandhoff” OR “Schindler” OR “Tay-Sachs”) ) | | |
| Boolean operator connecting search strings. | | AND | |
| Search Line 2:  NDIS String | ( {national disability insurance scheme} OR {national disability insurance agency} ) | | |
| Boolean operator connecting search strings. | | | AND |
| Search Line 3:  Age String | (Therap* OR Ergotherap* OR Dramatherap* OR Speech patholog*) OR (Counsel*) OR (Physiotherap*) OR (Physical PRE/0 Therap*) OR (Exercise PRE/0 Physiolog*) OR ((Personal OR Athletic OR Fitness) PRE/0 (Traine* OR Coac*)) OR (Dietitia* OR Dieteti*) OR (Podiatr* OR Chiropod*) OR (Social PRE/0 Wor*) OR (Nurs*) OR (Psycholog* OR Psychothera*) OR (Developmen* PRE/0 Educa*) OR (“Early childhood professional” OR “Early childhood professionals”) OR (Audiolog*) OR (Orthopt*) OR (“Therapy assistant” OR “Therapy assistants”) OR (Mentor*) | | |
